# "Familial" versus "Sporadic" intellectual disability: contribution of common microdeletion and microduplication syndromes

**DOI:** 10.1186/1755-8166-5-9

**Published:** 2012-01-29

**Authors:** Maryam Rafati, Elaheh Seyyedaboutorabi, Mohammad R Ghadirzadeh, Yaser Heshmati, Homeira Adibi, Zarrintaj Keihanidoust, Mohammad R Eshraghian, Gholam Reza Javadi, Jila Dastan, Alireza Mosavi-Jarrahi, Azadeh Hoseini, Marzieh Purhoseini, Saeed R Ghaffari

**Affiliations:** 1 Department of Medical Genetics, Tehran University of Medical Sciences, Tehran, Iran; 2 Comprehensive Genetic Center, Hope Generation Foundation, Tehran, Iran; 3 Department of Biology, Science and Research Branch, Islamic Azad University, Tehran, Iran; 4 Tehran Welfare Organization, Tehran, Iran; 5 Division of Pediatric Neurology, Department of Pediatrics, Tehran University of Medical Sciences, Tehran, Iran; 6 Department of Epidemiology and Biostatistics, School of Public Health, Tehran University of Medical Sciences, Tehran, Iran; 7 Department of Genetics, Science and Research Branch, Islamic Azad University, Tehran, Iran; 8 Gene Clinic, Tehran, Iran; 9 Department of Public Health and Social Medicine, Shahid Beheshti University of Medical Sciences, Evin, Tehran, Iran

**Keywords:** Familial Intellectual Disability, Mental Retardation, Common Microdeletion and Microduplication Syndromes, Hereditary

## Abstract

**Background:**

Interstitial Microdeletion and Microduplication syndromes have been proposed as a significant cause of sporadic intellectual disability (ID) but the role of such aberrations in familial ID has not yet been investigated. As the balanced chromosomal abnormalities commonly lead to the recurrent ID or multiple congenital anomalies, this study was designed to evaluate whether it was justified to investigate such aberrations in familial ID patients. Three hundred and twenty eight patients from 101 unrelated Iranian families with more than two ID patients in the first-degree relatives, have been investigated. Assessment of a panel of 21 common Microdeletion and Microduplication syndromes (CMMS) was carried out using Multiplex Ligation-Dependent Probe Amplification (MLPA) technique.

**Results:**

Among the families studied, 27.7% had 4-12, 35.6% had 3 and 36.6% had 2 affected individuals in the first-degree relatives. An autosomal dominant inheritance of Williams-Beuren syndrome (WBS) was detected in a family with no clinical suspicion of WBS. The prevalence of CMMS was therefore,0.99%.

**Conclusion:**

This is the first investigation of a panel of CMMS in a large sample set of "familial ID patients". The findings of this study showed the low prevalence of CMMSs in "familial ID" patients in spite of the significant contribution of such aberrations in "sporadic ID" which has a very useful practical impact by avoiding unnecessary diagnostic tests in "familial ID" patients.

## Background

Intellectual Disability, formerly Mental Retardation, is caused by heterogeneous genetic and non-genetic causes and affects 1-3% of the general population [1,2]. Regarding the heavy burden of ID, familial ID imposes much more burden on the affected families, society and health care system. Therefore, determining a specific genetic diagnosis facilitates both comprehensive medical care and accurate recurrence risk assessment for the family. As familial ID has a much lower incidence in western countries, its genetic basis has not been investigated to the extent of sporadic ID. Familial ID could be due to both chromosomal abnormalities and single gene disorders. In recent years the genetic basis of X-linked and autosomal recessive ID (ARID) has been thoroughly investigated and has lead to the identification of new genes and mutations [3-6]. However, no large scale study has so far been conducted to delineate the other underlying genetic causes of familial ID.

Chromosomal aberrations are considered to be the most frequent cause of unexplained developmental delay (DD), ID and multiple congenital anomalies (MCA) [7,8]. Emerging new molecular cytogenetic techniques like fluorescence in situ hybridization (FISH), multiplex ligation-dependent probe amplification (MLPA) and array-based whole genome screening has shown that the contribution of chromosomal aberrations in ID is not limited to the extent which was diagnosed by karyotyping alone (6-10%) and could be increased to as high as 20-30% using the mentioned methods [7,9,10]. As the balanced chromosomal abnormalities commonly lead to the recurrent DD/ID/MCA, this study was designed to evaluate whether it was justified to investigate such aberrations in familial ID patients with suggestive pedigree findings, via a stepwise approach to chromosomal abnormalities.

Interstitial Microdeletion and Microduplication syndromes account for 50-60% of the total submicroscopic chromosomal abnormalities detected in ID/MCA patients. Half of these are located in the regions of common Microdeletion and Microduplication syndromes (CMMS) including 22q11 deletion syndrome, Prader-Willi/Angelman, William-Beuren, Smith-Magenis, Miller-Dieker, 1p36 deletion syndrome and Soto syndrome [10].

The reported prevalence of CMMS has ranged from 5.8% to 9.2% in patients with sporadic ID based on the presence or absence of concurrent dysmorphism and/or MCA [10,11]. However, to the best of our knowledge, there are no reports of such investigations carried out on a large sample set of patients with familial ID.

Here, for the first time, we report the results of the assessment of CMMS in 101 families with familial ID in which cytogenetically visible abnormalities, Fragile-X syndrome and subtelomeric aberrations had already been excluded [12].

## Results

This study was carried out on a cohort of 328 individuals from 101 unrelated Iranian families with recurrent ID which included 32 affected parents and 296 affected individuals in the sibships. There were 185 males (56.4%) and 143 females (43.6%) in the population studied. After excluding the affected parents, the number and percentage of the male and female patients changed to 162 (54.7%) and 134 (45.3%) respectively. In 20 families one, and in 6 families both, of the parents were intellectually disabled.

The number of affected individuals with ID in the first-degree relatives of each family varied from 2 to 12 with a mean number of 3.15. More details are presented in table 1.

**Table 1:** Number of affected individuals in the first-degree relatives and the corresponding families.

Affected Individuals	Families (%)
2	37 (36.6%)

3	36 (35.6%)

4	18 (17.8%)

5	5 (4.9%)

6	2 (1.98%)

8	2 (1.98%)

12	1 (0.99%)

MLPA study using the probes specific for a panel of CMMS showed normal results for all but one investigated families. In the mentioned affected family, first step screening revealed deletion of ELN and LIMK1 genes in an 8-year old ID patient, suggestive of Williams-Beuren Syndrome. Further confirmatory studies showed also the deletion of FZD9 (Gene ID: 8326), STX1A (Gene ID: 6804), and CYLN2 (Gene ID: 269713) corresponding to WBS critical region genes. Follow-up parental investigations showed the same deletion in the 37-year old affected father.

## Discussion

Three hundred and twenty eight patients from 101 families with recurrent ID were investigated in this study, among which, one hereditary microdeletion syndrome was detected.

Demographic data showed that there was no notable excess of affected males (185:163) in the studied population due to the exclusion of Fragile-X syndrome as the most common cause of X-linked intellectual disability.

Cytogenetic abnormalities including interstitial microdeletion or microduplication syndromes are considered as the major cause of idiopathic sporadic ID [7,8]. In a retrospective investigation of 258 intellectually disabled and dysmorphic patients with normal Karyotype for a panel of CMMSs, Kirchhoff et al found 5.8% imbalances among the studied patients [11]. Further studies on another 170 patients with ID and dysmorphism, referred for Microdeletion study, revealed 17/80 (21.3%) and 7/90 (7.8%) imbalances in the patients with and without a suspicion of a specific Microdeletion syndrome respectively. On the other hand, Jehee et al found 9.2% Microdeletion and Microduplication syndromes in the patients with ID and multiple congenital anomalies [10]. Deletion of 22q11 region was the most common abnormality found, among them in 6 diagnosed patients, no clinical suspicion was proposed. The detected 0.99% prevalence of Microdeletion syndromes in "familial ID" patients studied here, was significantly lower than that of the previously reported studies on "sporadic ID" patients.

Microdeletion and Microduplication syndromes may occur as either isolated (sporadic) or inherited. The "mostly sporadic" nature of Microdeletion syndromes could be explained by the following two underlying molecular mechanisms. The first, which include most of the CMMSs, are mediated by Low Copy Repeats (LCRs) and therefore have consistent-sized deletions. The second group arise through some other mechanisms with varying-sized deletions [13]. The critical regions of most of the CMMSs consist of a single copy gene region flanked by repetitive sequences (LCRs). The deletions or duplications arise as a consequence of misalignment of these LCRs followed by unequal crossing over due to high similarity of them [14]. On the other hand, there are also several other underlying mechanisms leading to hereditary Microdeletion syndromes. These include those deletions or duplications inherited from clinically normal parents with balanced rearrangements, or those that are inherited from affected parents in which the initially "sporadic" deletion or duplication has passed through the subsequent generation.

The sample set of the present study is different from all of the previous investigations, as it is confined to just familial ID cases. Based on the mentioned feature, de novo CMMS were not expected to be detected among the studied population which could explain the low prevalence of detected CMMS.

Jehee et al have recently recommended using a combination of MLPA probe sets to detect chromosomal imbalances in patients with multiple congenital anomalies and mental retardation as a "valuable choice" for developing countries. They have used karyotyping in addition to MLPA analyses of subtelomeric rearrangements and CMMSs in MCA/ID patients. They have estimated, based on a decision analytic model, that this strategy could detect 76.45% of the total chromosomal abnormalities expected to be detected by karyotyping and whole genome array screening.

The patients studied here had already been investigated for cytogenetically visible chromosomal abnormalities, Fragile-X syndrome and subtelomeric rearrangements with no abnormality detected [12]. The MLPA probe sets used in this study for detection of CMMSs cover more syndromes than those investigated by Jehee et al. Therefore, all CMMSs and at least 76.45% of total chromosomal abnormalities have been ruled out in the families studied. It is therefore concluded that the ID present in the remaining families is either due to single gene disorders or chromosomal abnormalities outside the regions studied.

The diagnosed patients of the present report were not ever suspected for WBS during the long period of investigations, though other chromosomal or single gene disorders had been proposed clinically. This may be attributed to the heterogeneity in the phenotypes of the specific syndromes and their overlapping clinical features. Moreover, certain characteristics of the syndromes may not be fully manifested at the age the patient is studied.

Traditionally, Microdeletion syndromes were identified based on the serendipitous ascertainment of a patient with established clinical features and a chromosomal rearrangement detectable by G-banding or molecular cytogenetic techniques [8]. This "phenotype-first" approach has now somehow been replaced by the "genotype-first" approach. Screening methods such as MLPA or array-based genome screening have enabled us the identification of known or even novel imbalances in patients with ID and apparently nonspecific clinical features [13,15].

## Conclusions

This study is the first investigation of a large sample set of familial ID cases to evaluate the contribution of CMMS in familial ID which showed that it was not as much as what had been determined for sporadic ID. These findings show that CMMSs do not significantly contribute to familial ID.

Therefore, the findings of this and that of our previous study of subtelomeric rearrangements in these families suggest the low prevalence of chromosomal abnormalities in familial ID patients in spite of the significant contribution of such aberrations in sporadic ID. However, complementary investigations including the assessment of other genomic copy number changes by array-CGH, and the study of single gene disorders are recommended to delineate the underlying genetic causes in the remaining families.

## Methods

### Families and Patients

Since 2006, a number of families with recurrent ID registered in Tehran Welfare Organization, were undergone a stepwise approach which included karyotyping, assessment of Fragile-X syndrome, investigation of subtelomeric rearrangements and studying common Microdeletion and Microduplication syndromes. The families with positive findings in each step were excluded from subsequent investigations.

One hundred and one families which met the following criteria were included in this study: 1) The presence of at least two individuals in the first-degree relatives including the parents and siblings, affected with ID (diagnosed according to the standard definition of ID) or multiple congenital anomalies (MCA) in case of neonates that development could not yet be evaluated. 2) Normal results of the previous investigations including karyotyping, Fragile-X expansion mutation testing and assessment of subtelomeric rearrangements carried out in at least one of the affected individuals. Subtelomeric aberrations were studied by MLPA technique followed by subsequent confirmation of detected deletions or duplications by FISH method [12]. 3) No evidence of metabolic, neurodegenerative or other single gene disorders based on the available previous investigations including brain imaging and blood/urinary metabolic screening.

The study was approved by the Ethics Committee of Tehran University of Medical Sciences. The affected individuals, their parents and the normal siblings of each family, were all contacted to participate in this project. At the first visit, extended family pedigree was drawn, complete physical examination was conducted by a clinical geneticist and patients were photographed. Informed consents were obtained both from the patients and their parents or guardians. Anticoagulated blood samples were collected from the patients, the parents and the normal siblings of each individual family. Families had a pre-test genetic counseling and were informed of the objectives of this study and the details of the provided genetic tests. Pedigree information including clinical and paraclinical findings and details of all genetic evaluations were entered into an internally networked database. Post-test genetic counseling was scheduled for all families.

As it is generally accepted that all of the affected members in each individual family harbor the same mutation, one patient from each family was selected for assessment of Microdeletion and Microduplication syndromes based on the availability of the patients and parents and/or patients preferences. In case of detection of deletions or duplications, further subsequent studies were carried out on other affected individuals and their parents.

DNA was extracted from anticoagulated peripheral blood samples according to the standard phenol-chloroform DNA extraction protocol. The patients were screened for a panel of CMMSs listed in table 2, by MLPA technique, using SALSA P245 kit (MRC-Holland, Amsterdam, the Netherlands). The findings of screening tests were confirmed by two other SALSA MLPA kits (P064 and P096), covering more genes of CMMSs critical regions, when necessary. The details of regions detected by each kit can be found at http://www.mlpa.com.

**Table 2:** list of Microdeletion and microduplication syndromes investigated in this study.

Number	Microdeletion and microduplication syndromes	Genes
1	DiGeorge syndrome 22q11	CLDN5, AB-region GP1BB, AB-region SNAP29, CD-region

2	DiGeorge region 2, 10p15	GATA3 Hs.538604

3	Williams syndrome	ELN, exon 1 ELN, exon 20 LIMK1

4	Prader-Willi/Angelman	NDN SNRPN UBE3A

5	Cri du Chat syndrome, 5p15	TERT CRR9

6	Rubinstein-Taybi syndrome	CREBBP

7	Smith-Magenis syndrome	RAI1 LRRC48 LLGL1

8	Miller-Dieker syndrome, 17p	PAFAH1B1, ex 7 PAFAH1B1, ex 3

9	Langer-Giedion syndrome, 8q	TRPS1 EIF3S3

10	Sotos syndrome 5q35.3	NSD1, exon 17 NSD1, exon 22

11	NF1 microdeletion syndrome	NF1, exon 12 NF1, exon 20

12	Wolf-Hirschhorn 4p16.3	LETM1 WHSC1

13	WAGR syndrome	PAX6, exon 5

14	RETT syndrome, MECP2/Xq28 duplication	MECP2, exon 1 MECP2, exon 4

15	Phelan-Mcdermid syndrome (22q13)	SHANK3

16	1p36 deletion syndrome	TNFRSF4 GNB1 GABRD

17	2p16.1 microdeletion syndrome	FANCL REL

18	3q29 microdeletion syndrome	DLG1

19	9q22.3 microdeletion syndrome	TGFBR1 exon 7 TGFBR1 exon 8

20	15q24 microdeletion syndrome	SEMA7A exon 8 CYP1A1 exon 2

21	17q21.31 microdeletion syndrome	CRHR1, exon 8 MAPT, exon 11 MAPT, exon 13

### MLPA Analysis

Standard MLPA analysis was performed following the manufacturer's instructions. Since June 2011, new MLPA protocol (one-tube MLPA) was applied. Five-hundred (100 in one-tube MLPA) nanograms of genomic DNA was denatured and then hybridized with SALSA MLPA probemixes Following ligation, PCR was performed in a Gene Amp PCR system 9700 (Applied Biosystems, Foster City, CA, USA). Fluorescent amplification products were subsequently separated by capillary electrophoresis on an ABI 3130 Genetic Analyzer (Applied Biosystems, Foster City, CA, USA) and analysed using the Genemapper V4.0 software.

## Competing interests

The authors declare that they have no competing interests.

## Authors' contributions

SRG designed and initiated the study, monitored data collection and analysis for the whole study and drafted and revised the paper. He is guarantor. MR and ES contributed to the study design, implemented the technical parts, designed data collection tools, analysed the data, and drafted and revised the paper. MRG initiated the collaborative project and designed the data collection tools. YH, HA, ZTK, GRJ and JD helped with data collection and designed the data collection tools. MRE and AMJ analysed the data. AH and MP contributed to data collection and helped with technical parts. All authors have read and approved the final manuscript.

## References

[B1] Kriek M Knijnenburg J White SJ Rosenberg C den Dunnen JT van Ommen GJ Tanke HJ Breuning MH Szuhai K Diagnosis of genetic abnormalities in developmentally delayed patients: a new strategy combining MLPA and array-CGH Am J Med Genet A 2007 143 610 614 17318845 10.1002/ajmg.a.31593

[B2] Rimoin DLCM Pyeritz RE Korf BR Emery and Rimoin's Principles and Practice of Medical Genetics 2007 Fifth Pennsylvania: Churchill Livingstone Elsevier

[B3] Najmabadi H Hu H Garshasbi M Zemojtel T Abedini SS Chen W Hosseini M Behjati F Haas S Jamali P Deep sequencing reveals 50 novel genes for recessive cognitive disorders Nature 478 57 63 10.1038/nature10423 21937992

[B4] Kuss AW Garshasbi M Kahrizi K Tzschach A Behjati F Darvish H Abbasi-Moheb L Puettmann L Zecha A Weissmann R Autosomal recessive mental retardation: homozygosity mapping identifies 27 single linkage intervals, at least 14 novel loci and several mutation hotspots Hum Genet 129 141 148 10.1007/s00439-010-0907-3 21063731

[B5] Kaufman L Ayub M Vincent JB The genetic basis of non-syndromic intellectual disability: a review J Neurodev Disord 2 182 209 10.1007/s11689-010-9055-2 PMC2974911 21124998

[B6] Ropers HH Genetics of early onset cognitive impairment Annu Rev Genomics Hum Genet 11 161 187 10.1146/annurev-genom-082509-141640 20822471

[B7] Hochstenbach R van Binsbergen E Engelen J Nieuwint A Polstra A Poddighe P Ruivenkamp C Sikkema-Raddatz B Smeets D Poot M Array analysis and karyotyping: workflow consequences based on a retrospective study of 36,325 patients with idiopathic developmental delay in the Netherlands Eur J Med Genet 2009 52 161 169 10.1016/j.ejmg.2009.03.015 19362174

[B8] Shaffer LG Bejjani BA Torchia B Kirkpatrick S Coppinger J Ballif BC The identification of microdeletion syndromes and other chromosome abnormalities: cytogenetic methods of the past, new technologies for the future Am J Med Genet C Semin Med Genet 2007 145C 335 345 10.1002/ajmg.c.30152 17910076

[B9] Miller DT Adam MP Aradhya S Biesecker LG Brothman AR Carter NP Church DM Crolla JA Eichler EE Epstein CJ Consensus statement: chromosomal microarray is a first-tier clinical diagnostic test for individuals with developmental disabilities or congenital anomalies Am J Hum Genet 86 749 764 10.1016/j.ajhg.2010.04.006 PMC2869000 20466091

[B10] Jehee FS Takamori JT Medeiros PF Pordeus AC Latini FR Bertola DR Kim CA Passos-Bueno MR Using a combination of MLPA kits to detect chromosomal imbalances in patients with multiple congenital anomalies and mental retardation is a valuable choice for developing countries Eur J Med Genet 54 e425 432 10.1016/j.ejmg.2011.03.007 21457803

[B11] Kirchhoff M Bisgaard AM Bryndorf T Gerdes T MLPA analysis for a panel of syndromes with mental retardation reveals imbalances in 5.8% of patients with mental retardation and dysmorphic features, including duplications of the Sotos syndrome and Williams-Beuren syndrome regions Eur J Med Genet 2007 50 33 42 10.1016/j.ejmg.2006.10.002 17090394

[B12] Rafati M Ghadirzadeh MR Heshmati Y Adibi H Keihanidoust Z Eshraghian MR Dastan J Hoseini A Purhoseini M Ghaffari SR "Familial" versus "Sporadic" Intellectual Disability: Contribution of Subtelomeric Rearrangements Molecular Cytogenetics 2011 in press 10.1186/1755-8166-5-4 PMC3284400 22260313

[B13] Slavotinek AM Novel microdeletion syndromes detected by chromosome microarrays Hum Genet 2008 124 1 17 10.1007/s00439-008-0513-9 18512078

[B14] Schubert C The genomic basis of the Williams-Beuren syndrome Cell Mol Life Sci 2009 66 1178 1197 10.1007/s00018-008-8401-y 19039520 PMC11131529

[B15] Shaffer LG Theisen A Bejjani BA Ballif BC Aylsworth AS Lim C McDonald M Ellison JW Kostiner D Saitta S Shaikh T The discovery of microdeletion syndromes in the post-genomic era: review of the methodology and characterization of a new 1q41q42 microdeletion syndrome Genet Med 2007 9 607 616 10.1097/GIM.0b013e3181484b49 17873649

